# The Role of CTNNA1 in Malignancies: An Updated Review

**DOI:** 10.7150/jca.79236

**Published:** 2023-01-01

**Authors:** Jinhua Huang, Huihui Wang, Yuting Xu, Chunhua Li, Xinyue Lv, Xintong Han, Xiaochun Chen, Yu Chen, Zhiying Yu

**Affiliations:** 1 Department of Gynecology, Shenzhen Second People's Hospital/the First Affiliated Hospital of Shenzhen University Health Science Center, Shenzhen, 518035, Guangdong, China.; 2 College of Medicine, Shantou University, Shantou, 515041, Guangdong, China.; 3 Institute of Clinical Pharmacology, Anhui Medical University, Key Laboratory of Anti-Inflammatory and Immune Medicine, Ministry of Education, Anhui Collaborative Innovation Center of Anti-Inflammatory and Immune Medicine, Hefei, 230023, China.

**Keywords:** α-catenin, CTNNA1, tumorigenesis

## Abstract

Catenin alpha 1 (CTNNA1), encoding α-catenin, is involved in several physiological activities, such as adherens junction synthesis and signal transduction. Recent studies have suggested additional functions for CTNNA1 malignancies. This review systematically summarizes the varying functions of CTNNA1 in different tumors and briefly describes the diverse pathways and mechanisms involved in different types of tumors. CTNNA1 is abnormally expressed in leukemia and solid tumor such as cancers of digestive system, genitourinary system and breast, and it's related to the occurrence, development, and prognosis of tumors. In addition, the possible physiological processes involving CTNNA1, such as methylation, miRNA interference, or regulatory axes, similar to those of CDH1, SETD2, and hsa-miR-30d-5p/GJA1 are also summarized here. The precise mechanism of CTNNA1 in most cancers remains uncertain; hence, additional pre-clinical studies of CTNNA1 are warranted for potential early tumor diagnosis, prognosis, and treatment.

## Introduction

Cancer is one of the most life-threatening diseases 1. Catenin alpha 1 (CTNNA1), located on the chromosome band 5q31 2, encodes α-catenin, an important protein in the E-cadherin-catenin complex 3. This complex participates in building and maintaining a multicellular organism and adjusts or participates in various cellular functions, including signal transduction, cell growth, differentiation, site-specific gene expression, morphogenesis, immune function, cell movement, wound healing, and inflammation. The E-cadherin-catenin complex has several functions in tumor cells, such as invasion and metastasis 4.

CTNNA1 is widely expressed in normal human tissues 5; recent studies revealed that deletion of CTNNA1 expression activates tumor necrosis factor alpha converting enzyme (TACE), which increases transforming growth factor alpha (TGF-α) activity and promotes cell proliferation and wound healing 6. In addition, α-catenin is one of the substrates of cellular autophagy, a switch of Yes-associated protein 1 (YAP1)-transcriptional co-activator with PDZ-binding motif (WWTR1/TAZ), which acts as a negative regulator in this pathway. Conversely, autophagy perturbation activates YAP1-WWTR1/TAZ by promoting the degradation of α-catenin and thereby cell growth 7. α-Catenin maintains a certain stretch and open state in response to vinculin, which modulates YAP1 and regulates cell proliferation; hence, α-catenin deficiency or deletion weakens adherens junction (AJ), which allows YAP1 to freely enter and exit the nucleus, relieving vinculin of its silencing effect on the stationary phase of the stem cell cycle, thus promoting cell proliferation 8. CTNNA1 have also been revealed to the development and progression of numerous malignancies. This paper reviews the relationship between CTNNA1 and various malignancies and presents the possible pathogenic mechanism of CTNNA1 in malignancies (Table 1).

## Role of CTNNA1 in intestinal cancer

Immunohistochemistry assays reveal that CTNNA1 expression is decreased in colorectal cancer (32/82) compared with that in normal colorectal mucosa and colorectal adenoma (8/10). Meanwhile, CTNNA1 expression decreases or is very low in lymph node metastases (6/10). This suggests that low CTNNA1 expression correlates with lymphatic metastasis, poor differentiation, and poor prognosis of colorectal cancer 9. Decreased CTNNA1 expression is also found in colon cancer 10. Three-dimensional culture technology has shown that CTNNA1 represses cell adhesion and invasion but has no impact on the sensitivity of colorectal cancer cells to chemoradiotherapy 11.

**Figure 1:**
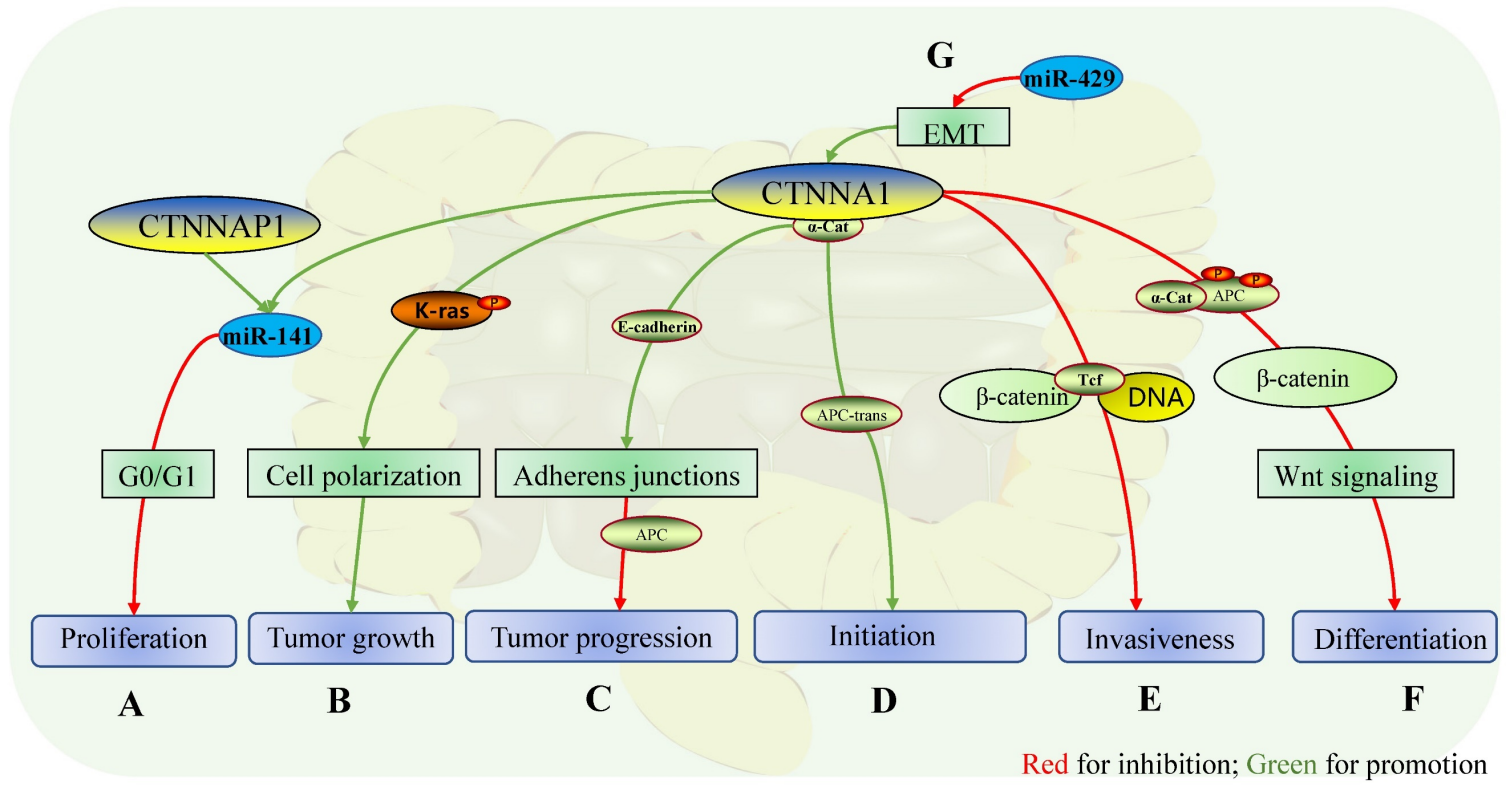
** Role of CTNNA1 in intestinal cancers.** The mechanisms of CTNNA1 involvement in cancer of the intestinal tract: **A** In colorectal cancer cells, the homologous gene of CTNNA1, CTNNAP1 regulates the expression of CTNNA1 by competing with endogenous RNA (Mir-141) *in vitro*, while the overexpression of CTNNA1 or CTNNAP1 inhibits cell proliferation during G0 or G1 phase of the cell cycle; **B** CTNNA1 is expressed in phospho-/dephosphorylatable K-Ras cells to maintain cell polarity, which facilitates tumor growth, although the specific mechanism is unclear; **C** In advanced colorectal cancer, CTNNA1 promotes the suppressive effect of APC by maintaining the function of E-cadherin on cell adhesion; **D** In a group of intestinal adenomas, somatic recombination leads to cis to trans-APC mutations, which results in the retention of CTNNA1 expression, which promotes the initiation of intestinal adenomas; **E** In colon cancer, α-catenin represses transcription of the invasive phenotype by inhibiting the binding of the β-catenin-Tcf complex to DNA; **F** In colon cancer, α-catenin stabilizes its attachment to APC and protects phosphorylation determinants, which promotes the ubiquitination and protein hydrolysis of β-catenin, and downstream inhibits the Wnt pathway and suppresses endodermal differentiation; **G** In colorectal cancer, Mir-429 improves the expression of CTNNA1 by reversing the EMT mediated by TGF-β and plays a role in inhibiting tumor growth and invasion.

Almost all intestinal adenomas from familial adenomatous polyposis (FAP) mouse lines that carry a truncation mutation at codon 580 in adenomatous polyposis coli (Apc580D) have both CTNNA1 and adenomatous polyposis coli (APC) gene deletions. Furthermore, loss of heterozygosity (LOH) of CTNNA1 is obtained in the cis configuration of mutant APC, a mutant construct of APC in intestinal adenoma, except for one cell line with almost normal α-catenin expression. Somatic recombination alters the cis configuration of mutant APC and CTNNA1, resulting in wild-type CTNNA1 retaining the trans configuration with APC; this configuration may result in somatic recombination between APC and CTNNA1 sites in a cell line, possibly before APC LOH. This retains CTNNA1 expression and prevents the decrease of α-catenin, despite LOH. Under these conditions, it is believed that CTNNA1 retention supports the early initiation of intestinal adenoma in the absence of APC, while inhibition of intestinal adenoma only occurs when CTNNA1 is in the cis configuration with APC (Fig. 1E) 12. However, CTNNA1 plays an inhibitory role in advanced colorectal cancer, which may be related to the Wnt and K-Ras pathways, although the specific mechanism remains unknown.

Truncation mutations in APC lead to dysregulation of Wnt signaling and cell adhesion changes, while CTNNA1 is essential for maintaining E-cadherin on the cell surface (Fig. 1D) 13, suggesting that CTNNA1 can promote suppression of APC in tumors. In contrast, the absence of CTNNA1 in most intestinal adenoma cells is accompanied by the lack of APC, which cannot infer the contribution of CTNNA1 deletion to the formation of intestinal adenoma. On the contrary, in intestinal adenoma cell lines expressing α-catenin with mutated APC, CTNNA1 promotes the initiation of intestinal adenoma. The two aspects seem contradictory, and the specific mechanism of CTNNA1 in intestinal adenomas is not fully understood, and hence, further investigation of the relationship between CTNNA1 and K-Ras, phosphatase and tensin homolog (PTEN) 14, Wnt, MAPK/PI3K, and TGF-β 15 in colorectal cancer is warranted.

In sporadic colorectal cancer, CTNNA1 and CTNNB1 mutations are associated with younger diagnosis age (< 50 y), while patients with colorectal cancer (CRC) with CTNNA1 mutations have considerably increased lymph node metastasis 16. CTNNA1 can disrupt the interaction between the β-catenin-Tcf complex and DNA *in vitro*, which inhibits the transcription of genes involved in tumor cell invasion 17, suggesting that CTNNA1 and the Wnt pathway are germane (Fig. 1F). The stable association of CTNNA1 with APC protects the phosphodegron (proteins in the cell-cell attachment complex that are recognized by the E3 ubiquitin ligase complex), promotes β-catenin ubiquitination and proteolysis, and inhibits the Wnt pathway (Fig. 1G) 18. In addition, large-scale sequencing of appendiceal goblet cell carcinoma and primary colorectal adenocarcinoma identified gene mutations related to the Wnt pathway, including that of CTNNA1 19. Therefore, CTNNA1 plays a role as a tumor suppressor gene in colorectal cancer by inhibiting the Wnt pathway.

K-Ras expression slightly increases in DLD-1(human colorectal cancer cell line grown in medium containing non-essential amino acids), while CTNNA1, β-catenin, and E-cadherin show a significant decrease compared with those in control Coca-2 (human colorectal cancer cell line grown in medium without non-essential amino acids) 20, suggesting that K-Ras may interact with the E-cadherin-catenin complex 20. CTNNA1 is highly expressed in DLD-1 cells with phosphor-/dephosphorylatable K-Ras, a necessary phenotype for maintaining epithelioid morphology and tumor growth, which may be associated with maintaining cell polarization and cell invasion. On the contrary, CTNNA1 expression decreases in cells with phosphomimetic or non-phosphorylatable K-Ras, and cell adhesion decreases accordingly 21.

Therefore, CTNNA1 may maintain the polarization state of cells in colorectal cancer, which in turn maintains the epithelioid morphology of cancer cells via regulating K-Ras phosphorylation (Fig. 1C).

MicroRNA is a non-coding endogenous RNA containing approximately 22 nucleotides that plays an important role in regulating gene transcription in many biological processes 22. Abnormal miRNA expression is associated with various types of cancer 23. For instance, miR-429 inhibits cell metastasis and invasion and reverses TGF-β-associated epithelial-to-mesenchymal transition (EMT) in colorectal cancer, and can reverse the changes in related marker genes induced by TGF-β in EMT, including CTNNA1 (Fig. 1A) 24. The allelic pseudogene of CTNNA1, CTNNAP1, competes with endogenous RNA (miR-141) to regulate CTNNA1 expression, which inhibits tumor growth and cell proliferation in the G1 or G0 phase of cell division *in vitro* and *in vivo*. Furthermore, overexpression of CTNNA1 or CTNNAP1 inhibits cell proliferation and tumor growth at G0 or G1 (Fig. 1B) 25. Thus, CTNNA1 may act as a tumor suppressor gene by interacting with miRNAs.

Taken together, CTNNA1 plays a dual role in intestinal tumors-suppressive and oncogenic. We hypothesize that the suppressive role of CTNNA1 may be through the inhibition of transcription of certain genes associated with carcinoma cell invasion or the inhibition of certain oncogenic pathways, such as Wnt or PTEN, as well as the inhibition of cell invasion in cell division, which may be regulated by miRNAs. As for the oncogenic role, it may be related to the mutational deletion of CTNNA1 with certain oncogenes or changes in the methylation levels. Therefore, the mutation status of CTNNA1 is essential for intestinal cancer.

### Role of CTNNA1 in breast cancer

**Figure 2:**
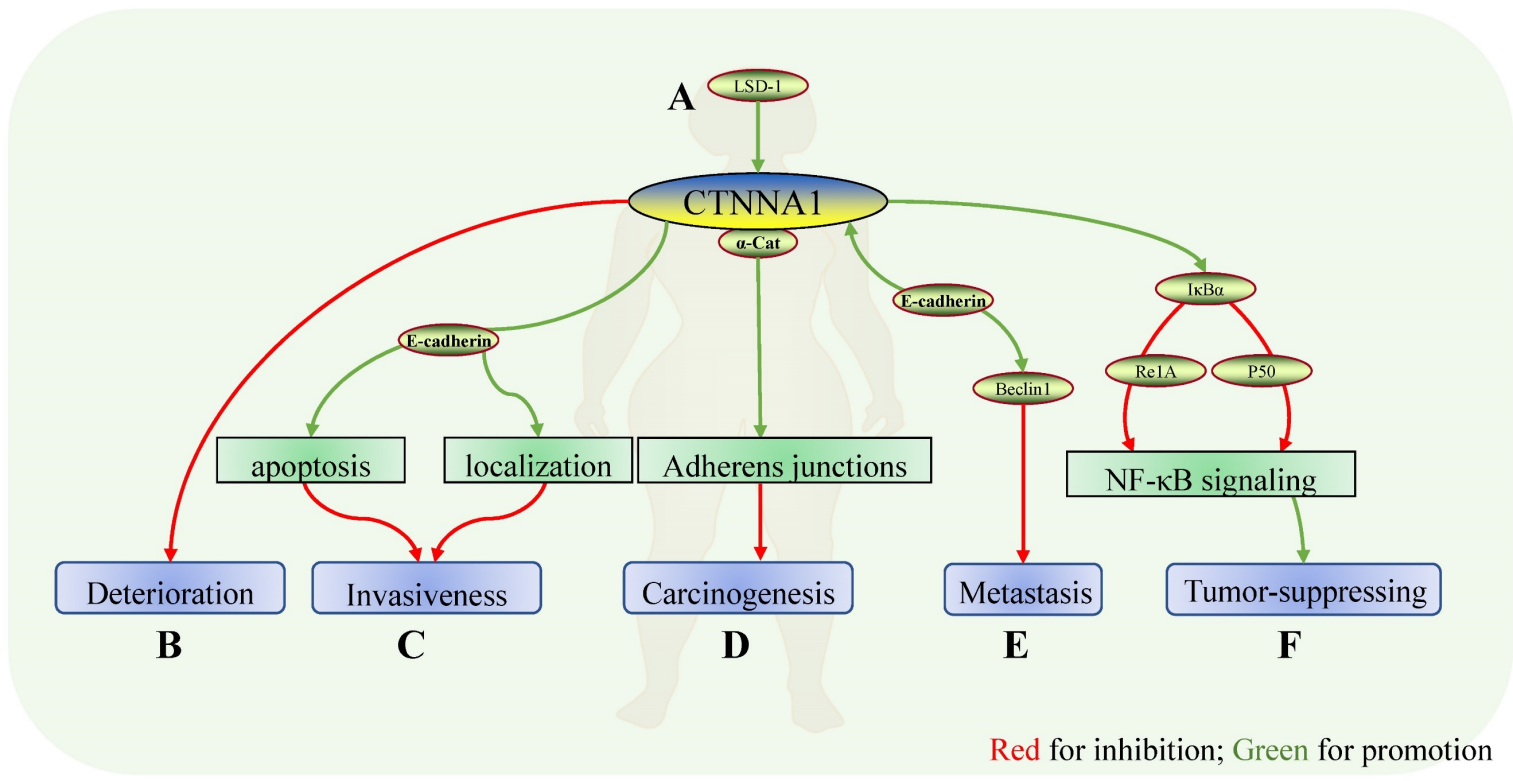
** Role of CTNNA1 in breast cancer.** Mechanisms of CTNNA1 involvement in breast cancer:** A** In a luminal breast cancer mouse model, LSD1 ensured the expression of cell adhesion molecules, including CTNNA1; **B** CTNNA1 expression showed a significant decrease in breast carcinoma *in situ*, breast cancer with metastasis, and ductal carcinoma, suggesting that CTNNA1 might be negatively correlated with the deterioration of breast cancer. **C** In mouse models and human breast cancer tissues with normal E-cadherin, α-catenin maintained E-cadherin localization and promoted apoptosis. The absence of α-catenin induced atypical localization of E-cadherin and apoptosis resistance which might depend on Rho/ Rock-dependent actomyosin contractility and led to the invasive characteristics of tumor cells. **D** Deletion of α-catenin resulted in the loss of cell-cell adhesion and increased the tumorigenic properties of cells *in vitro*. **E** Conditional knockdown of CTNNA1 and CDH1 reversed the inhibition of Beclin1 on the proliferation and metastasis of cancers. At the same time, enhancing the expression of Beclin1 increased the expression of CTNNA1 and CDH1. **F** α-catenin bound to IκBα and inhibited its ubiquitination and protease hydrolysis to localize Re1A and p50 on the nucleus, thereby reducing the expression of TNF-α, Re1B, and IL-8, and inhibiting the NF-κB pathway. In CDH1-negative basal-like breast cancer, deletion of α-catenin reversed this inhibition and derepressed carcinoma.

α- and β-catenin progressively decrease in pure lobular carcinoma *in situ* (LCS), lobular carcinoma *in situ* with concurrent invasive lobular carcinoma (ILC), and LCIS with concurrent infiltrating ductal carcinoma (IDC)/ductal carcinoma *in situ* (DCIS) (Fig. 2B) 26. Meanwhile, the expression and distribution of TWIST protein in these tissues are progressively increased. This suggests that the progressive loss of functional CTNNA1 and CTNNB1 plays a role in the progression and invasion of lobular carcinoma to lobular invasive carcinoma *in situ*, which may be related to a progressive increase in TWIST expression 26. Deletion of the cadherin-catenin complex containing α-catenin plays a role in the carcinogenesis and development of breast lobular carcinoma (Fig. 2D).

α-catenin reduction coincides with lobular carcinoma progression *in situ*, which deteriorates to the more aggressive, ductal carcinoma, suggesting that breast cancer α-catenin levels may influence the degree of progression. A CTNNA1 deletion in tumor tissues and subsequent metastatic lesions is consistent with this. Parallel DNA sequencing of four tumor samples reveals two overlapping deletions on chromosome 5 in all three tumor samples, including CTNNA1, suggesting that CTNNA1 deletion may be involved in metastasis 27. Deletion of α-catenin in mouse models and human breast cancer tissues with normal E-cadherin expression induces atypical localization and apoptotic resistance of E-cadherin, which relies on Rho/Rock-dependent actomyosin contractility (Fig. 2C). This demonstrates that α-catenin inactivation leads to the lobular characteristic and invasive behavior; therefore, α-catenin is a presumed tumor suppressor molecule in basal-like breast cancer 28. Meanwhile, intravenous injection of α-catenin carried by carbonate apatite nanoparticles reduces breast cancer tumor volume 29. In conclusion, CTNNA1 may inhibit breast cancer deterioration and promote cell apoptosis and tumor enlargement, which may be related to Rho/Rock.

The expression of CTNNA1 and CDH1 is related to the estrogen-positive phenotype in grade III breast ductal carcinoma 30. Meanwhile, retention of CDH1 and CTNNA1 is related to shorter relapse-free survival (RFS) and overall survival (OS) 30. Subsequent studies on breast ductal carcinoma and axillary lymph node metastases show decreased α-catenin levels in the primary lesions of breast ductal carcinoma. In addition, CTNNA1 re-expression obtained in the corresponding lymph node metastases is a common event in breast ductal carcinoma and plays a central role during the establishment of metastasis. This is different from the phenomenon in which positive CTNNA1 expression is detected in metastatic lesions of colorectal cancer, as outlined above. Therefore, the specific function of CTNNA1 may be dissimilar in different tumors. CTNNA1 and CDH1 are associated with shorter survival in grade III breast ductal carcinoma, and strong expression of adhesion molecules in metastatic tumors may be associated with more aggressive cancer phenotypes 31. Whole genome and transcriptome sequencing of triple-negative breast cancer show many mutated genes (including CTNNA1) with potentially metastatic implication 32. Breast cancer cells lacking CTNNA1 completely lose their ability to adhere to one another, which increases carcinogenesis *in vitro*, indicating that CTNNA1 deletion serves an important role therein 33.

CTNNA1 forms a stable structure with IκBα and inhibits its ubiquitination and binding with protease. It prevents localization of Re1A and p50 to the nucleus, which reduces the expression of TNF-α, Re1B, and IL-8. In this way, CTNNA1 inhibits the NF-κB pathway. CTNNA1 expression significantly decreases in basal-like breast cancer with negative CDH1, which shows a negative correlation with the level of TNF-β and Re1B. This demonstrates that the NF-κB pathway is activated in basal-like breast cancer with negative CDH1 due to the loss of CTNNA1 (Fig. 2F) 34.

CRISPR/Cas9 knockdown of CTNNA1 and CDH1 reverses the inhibitory effect of Beclin-1 on breast cancer cell proliferation. Enhanced Beclin-1 expression increases cell surface localization of E-cadherin complexes (composed of E-cadherin, CTNNA1, and β-catenin) 35. Therefore, CTNNA1, as a constituent molecule of the E-cadherin complex, promotes the suppressive function of Beclin-1 (Fig. 2E).

Induced loss of LSD1 in luminal cells significantly increases lung metastasis in a luminal breast cancer mouse model. However, loss of LSD1 reduces the expression of cell adhesion molecules (including CDH1 and CTNNA1); furthermore, TRIM37, encoding histone H2A ubiquitin ligase, is upregulated in either LSD1 or GATA3 knockdown cell lines, leading to the aforementioned inhibition 36. This suggests that CTNNA1 plays a role in the anti-cancer effect of LSD1 in luminal breast cancer, while TRIM37 inhibits this anti-cancer effect (Fig. 2A).

A very small number of breast cancer cases have somatic mutations or homozygous deletions of CTNNA1 (14/1101), accompanied by homologous recombination pair deletions with high similarity to BRCA1/2, according to The Cancer Genome Atlas (TCGA) data from 1101 breast cancer cases 38. This suggests that somatic mutations or homozygous deletions of CTNNA1 may affect sensitivity to treatments such as platinum salts or poly ADP ribose polymerase (PARP) inhibitors.

The loss of CTNNA1 releases the suppression of the NF-κB pathway in breast cancer, while a CTNNA1 deletion attenuates the tumor suppressive effect of Beclin-1. Loss of LSD1 reduces the level of CTNNA1 and other cell adhesion molecules, suggesting that CTNNA1 also plays a role in the cancer suppressive function of LSD1. Notably, interference of CDH1, also an inhibitor of cancer, may be involved in the cancer inhibitory effect of CTNNA1. Future studies should consider excluding the interference of CDH1 and demonstrating the interaction between CTNNA1 and CDH1.

### Role of CTNNA1 in hematologic malignancies

Analysis of all 297 patients identified 30 differentially expressed genes, including CTNNA1 37. Molecular cytogenetic fluorescence *in situ* hybridization techniques and array comparative genomic hybridization were used to detect partial chromosomal deletions and genomic deletions in acute myeloid leukemia (AML) cells, including those of CTNNA1 38. Twenty-two percent of 386 therapy-related AML or MDS cases have institutional abnormalities on chromosome 5. Haploinsufficiency of RPS14, EGR1, APC, NPM1, and CTNNA1 genes on chromosome 5q are associated with MDS/AML pathogenesis 39.

An analysis of copy number aberrations and copy-neutral loss of heterozygosity in a case of MDS with normal karyotypes detected a minimal 5q deletion, including CTNNA1 40, 41. The CTNNA1 promoter is inhibited by phosphorylation and histone deacetylation in a cell line carrying the 5q31 deletion, HL-60. Re-expression of CTNNA1 reduces HL-60 proliferation and promotes apoptosis. The 5q deletion more likely leads to a loss of CTNNA1 in MDS or AML, compared with those without a 5q deletion or normal stem cells, which may be accomplished through DNA methylation or histone deacetylation 42. CTNNA1 hypermethylation is more common in AML patients with a 5q deletion than in AML patients without a 5q deletion, while no methylation occurs in the other 5q genes. Hypermethylation in MDS is only found in high-risk MDS, not in low-risk MDS, suggesting that CTNNA1 methylation may be related to the progression of MDS into AML. A chromosomal inhibition marker (H3K27me3) is detected at the CTNNA1 promoter in AML, or primary leukemia, with CTNNA1 inhibition, and the most inhibited state is associated with CTNNA1 hypermethylation. Although there is a subset of AML without CTNNA1 promoter methylation, CTNNA1 expression is the lowest in AML and MDS accompanied by CTNNA1 methylation. Therefore, inhibition of CTNNA1 expression may be influenced by its methylation status 43. Leukemia cases with normal cytogenetics were more likely to have tumor suppressor gene (TSG) methylation (including CTNNA1) compared with AML or MDS cases with abnormal karyotypes 44. Methylation-specific PCR (MSP) shows that AML patients with CTNNA1 hypermethylation show significant reduction in CTNNA1 transcription 45. The authors also reported an insignificant shorter OS of AML patients with CTNNA1 methylation than those without (6 vs 9 months; P = 0.681) 45. A similar study evaluated CTNNA1 methylation by quantitative MSP, wherein the samples were selected more strictly, with over 50% bone marrow blasts performed 46. Twenty-five percent of AML patients show CTNNA1 hypermethylation, which mostly coexists with ASXL1 and RUNX1 variants, and is associated with unfavorable karyotypes. These studies, although similar, reported different results; however, the following aspects may result in unmeasured errors in the detection data of samples with under 50% of bone marrow blasts: bone marrow aspiration site, specimen transportation mode, cryopreservation time, and repeated freeze-thaw cycles. Thus, AML with CTNNA1 hypermethylation is more serious and has a shorter RFS and OS. In addition, multivariate analyses show that CTNNA1 hypermethylation is an independent factor for poor prediction of RFS, but not for OS 46.

PTEN regulates CTNNA1 expression by modifying the ratio of P42 CCAAT/enhancer binding protein (C/EBP) to P30 C/EBP, thereby affecting the progression of bone marrow dysplasia and AML. Retention of CTNNA1 proximal promoter binding to P30 CEBP (low P42/P30 ratio) inhibits CTNNA1 expression by inhibiting H3K27me3 to recruit more polycomb complex 2 (PRC2). On the contrary, binding to P42 CEBP (high P42/P30 ratio) relieves the inhibition and promotes CTNNA1 expression. Knockdown of PTEN in wild zebrafish and mice causes a decrease in the expression of wild-type PTEN product (P42) and α-catenin, inducing bone marrow dysplasia 47. Therefore, CTNNA1 expression is also regulated by the ratio of P42/P30 in a PTEN-dependent manner.

**Figure 3:**
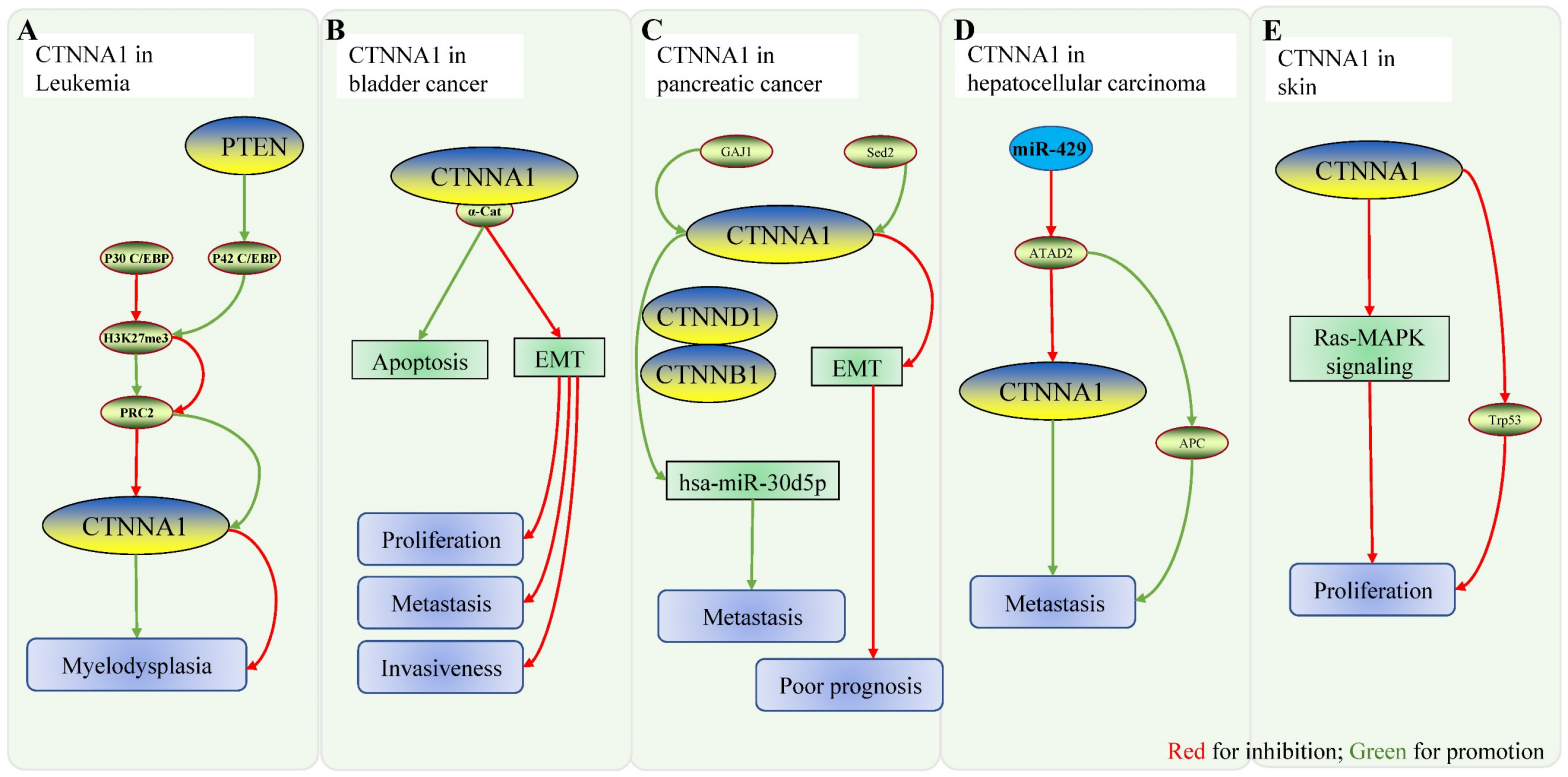
** Role of CTNNA1 in other malignancies. A** Mechanistic model of CTNNA1 involvement in hematological cancers: the ratio of P42/P30 regulates the expression of CTNNA1. At low ratios, p30C/EBP binds to the promoter of CTNNA1, represses H3K27me3 at the promoter, and recruits more PRC2 to repress CTNNA1 expression, leading to myeloproliferative disorders. When the ratio is high, p42C/BEP unbinds p30C/EBP to the CTNNA1 promoter, enhancing CTNNA1 expression and suppressing myeloproliferative disorders. **B** Role of CTNNA1 in bladder cancer: CTNNA1 promotes cell apoptosis and inhibits EMT, thereby inhibiting cell proliferation, tumor invasion, and metastasis. **C** Role of CTNNA1 in pancreatic cancer: ① GAJ1 enhances hsa-Mir-30d5p by promoting the expression of CTNNA1, CTNNB1 and CTNND1, which leads to the metastasis of pancreatic cancer. ② In pancreatic ductal carcinoma, Setd2 ablation downregulates CTNNA1 expression through impaired epigenetic inheritance, which then enhances EMT, ultimately resulting in poor prognosis. **D** CTNNA1 in hepatocellular carcinoma. mir-429 downregulates ATAD2, while overexpression of ATAD2 downregulates CTNNA1 and upregulates APC, which positively correlates with tumor metastasis. Mir-429 downregulates ATAD2, while overexpression of ATAD2 downregulates CTNNA1 and upregulates APC, which positively correlates with tumor metastasis. **E** CTNNA1 in skin: *In vivo*, deletion of CTNNA1 enhances the RAS-MAPK pathway and activates Trp53, which promotes cell proliferation.

The reduced expression of CTNNA1 in AML is associated with its methylation during cell apoptosis. Combined with the existing literature, we speculate that this may be related to the methylation of H3K27me3 at the promoter of this gene; PTEN has been demonstrated to promote the expression of CTNNA1 and thus inhibit myeloproliferation. The role of other pathways, such as JMJD3 and HOX 48, associated with CTNNA1 still needs to be further validated (Fig. 3A).

### Role of CTNNA1 in gastric cancer

CDH1 is a suppressor gene of gastric cancer, Although the variation in CTNNA1 was not initially noticed since it did not induce carcinogenesis 49, further studies have shown that CTNNA1 is indeed a susceptibility gene for gastric cancer 50. Germline mutations of CTNNA1 play a role in a few gastric cancer cases or families 50-56. A cohort study of 207 patients with gastric cancer (161 families) detected one pathogenic CTNNA1 variant in two patients of the same family 57, demonstrating that CTNNA1 is most likely a suppressor gene of gastric cancer. Subsequent experiments have reinforced this conclusion 58. A germline mutation of CTNNA1 was identified in one family without an obvious CDH1 mutation, followed by silencing of residual CTNNA1 in biopsies under gastroscopy 51 in two members of a families affected by gastric cancer and four members of families containing signet-ring cells, a histopathological indicator of gastric cancer based on microscopic features with a poor prognosis 59. Repeated observation of the cohort of gastric cancer patients without CDH1 mutations discovered CTNNA1 mutations (2/28), supporting the hypothesis that pathogenic CTNNA1 variants are the rare causes of gastric cancer 60. Next-generation sequencing of unexplained young or familial gastric cancer cases without CDH1 mutations revealed that CTNNA1, MYD88, and MAP3K6 had deleterious mutations associated with gastric cancer. A gastroscopic screen of 40 first-degree relatives of patients with hereditary diffuse gastric cancer (HDGC) without CDH1 mutations discovered signet-ring cell carcinoma in four patients from one family diagnosed with germline mutations in CTNNA1 61. A retrospective study of CDH1 mutant-negative patients suspected of HDGC found loss of CTNNA1 in diffuse gastric cancer, suggesting that CTNNA1 may be a new susceptibility gene for gastric cancer 62.

Immunohistochemical and PCR analyses showed that CTNNA1 mRNA was decreased or absent in 13/19 gastric mucosa from gastric cancer cases and their first-degree relatives (including 7 cases with CTNNA1 loss and 6 with decreased expression), which were significantly different from that of normal gastric mucosa. Additionally, decreased CTNNA1 expression was discovered in the gastric and antrum mucosa of first-degree relatives (11/18 of the gastric body mucosa and 4/18 of the antral mucosa). The decrease or downregulation of CTNNA1 mRNA in the gastric mucosa was associated with Helicobacter pylori infection (P < 0.05) 63. It seems that CTNNA1 correlates with familial aggregation of gastric cancer, which is supported by the gastroscopic screening of 40 first-degree relatives of CDH1 mutation-negative HDGC patients revealing signet-ring cells in four patients from one family 61. Nine new CTNNA1 mutations were identified in diffuse gastric cancer from 38 patients with sporadic diffuse gastric cancer and 10 patients with HDGC. Most of the patients had stage III gastric cancer, suggesting that CTNNA1 mutations may correlate with gastric cancer pathogenesis and even advanced cancer 64. Although a close relationship between CTNNA1 mutations and gastric cancer has been established, and some diagnosis and treatment guidelines have been proposed 50, the specific effect of CTNNA1 on gastric cancer pathogenesis remains elusive.

At present, pathogenic mutations in CTNNA1 explain < 2% of the HDGC family, while previous studies have detected at least 25 mutations in CTNNA1 in gastric cancer 65. Most of these studies remain at the genetic level; hence future studies should investigate the specific mechanisms of CTNNA1 affecting gastric cancer, including the APC, Wnt, PTEN, and K-Ras pathways involved in the same digestive tract tumors of the intestine 66.

### Role of CTNNA1 in gynecological cancers

Positive expression of CDH1, CTNNA1, and CTNNB were associated with good prognosis for endometrial cancer 67 and correlated with FIGO stages I-II (P = 0.02). Negative CDH1 expression was associated with FIGO stage III and non-endometrial carcinoma (NEEC), while the specific role of CTNNA1 or CTNNB1 remains to be elucidated. Knockdown of CDH1 in mouse embryos resulted in structural disruption of endometrial epithelial cells and extinction of endometrial glands in newborn mice. This may be due to disruption of CTNNA1 or CTNNB1 expression disrupting the Wnt pathway and Hox genes in the newborns and inducing proliferation of abnormal epithelial cells. The uterus of these mice had the aggressive histological features of endometrial cancer. In combination with the methylation of CTNNA1 in endometrial cancer specimens 68, whether the CTNNA1 gene is responsible for the invasive features of the mouse uterus in this assay remains to be determined 69.

Five hypermethylated genes (CD97, CTNNA1, DLC1, CD97, and HAPLN2) and three hypomethylated genes (LAMA4, LPP, and MFAP4) are significantly associated with poor recurrence-free survival of ovarian cancer tissues from TCGA (n = 391) using multivariate Cox regression analysis. After adjustment for age and stage, patients with at least two hypermethylated sites in CTNNA1, DLC1, or MFAP4 were significantly correlated with a worse progression-free survival 70. As a novel cancer biomarker 71. CTNNA1 hypermethylation could be an independent prognostic factor for poor RFS in AML 46; therefore, genome sequencing or immunohistochemistry may be used to investigate the role of CTNNA1 methylomics in ovarian cancer or the specific impact of prognoses related to chemotherapy or immunosuppressive therapy.

### Role of CTNNA1 in bladder cancer

Decreased expression of CTNNA1 and PTEN correlated with a high pathological stage of bladder cancer through immunohistochemical analysis of 133 cases of bladder cancer (P = 0.01) 72. An analogous conclusion was reached using qRT-PCR and western blot analysis of bladder cancer tissues and cell lines 73. Various pathways have been identified in bladder cancer exosomal proteins, including that of CTNNA1 74. CTNNA1 enhances the expression of CDH1 and inhibits the expression of CDH2, SNAI1, MMP2, and MMP9 in bladder cancer cells. These results suggest that CTNNA1 may inhibit cell proliferation, migration, invasion, and EMT in bladder cancer cells, while promoting cell apoptosis 72. The discovery that low CTNNA1 expression is associated with a higher degree of bladder cancer progression, accompanied with the discovery that the CTNNA1 pathway exists in the exosomes of bladder cancer cells, will facilitate future studies on CTNNA1 in other cancer types (Fig. 3B).

### Role of CTNNA1 in pancreatic cancer

A group of pancreatic cancer cells with high metastasis had over 40 differentially expressed genes (including CTNNA1) with liver metastasis tissues of pancreatic cancer; this was three times more than untreated cells 75. This suggests that CTNNA1 may be associated with tumorigenesis or metastasis in pancreatic cancer. The decrease or loss of expression of SETD2 predicted a poor prognosis of pancreatic ductal carcinoma. SETD2 is a suppressor gene of K-Ras-associated pancreatic cancer, and its loss in acinar cells promotes K-RAS-induced pancreatic ductal reprogramming through epigenetic dysregulation of Fbxw7. SETD2 ablation in pancreatic cancer cells also enhances EMT through impaired epigenetic regulation of CTNNA1 76. Meanwhile, the hsa-miR-30d-5p/GJA1 axis is a likely pathway involved in pancreatic cancer metastasis 77. Differential expression analysis, survival analysis, target gene prediction, pathway enrichment analysis, cross-linking analysis, and correlation analysis were used to determine gene expression profiles of metastatic pancreatic cancer cell, M8, and its parent cell, BxPC. CTNNA1, CTNNB1, and CTNND1 are key molecules potentially involved in pancreatic cancer metastasis mediated by hsa-miR-30d-5p/GJA1. Moreover, survival analysis shows that pancreatic cancer patients with high expression of CTNNA1, CTNNB1, or CTNND1 have a poor prognosis. These results suggest that GJA1 may contribute to pancreatic cancer metastasis by enhancing the expression and function of CTNNA1, CTNNB1, and CTNND1 (Fig. 3C) 77.

The inhibitory effect of CTNNA1 in bladder cancer was implicated in enhancing CDH1 expression and inhibiting EMT. In pancreatic cancer, ablation of SETD2 caused the deletion of CTNNA1, thereby mediating the enhancement of EMT, which strongly suggests that CTNNA1 plays a role in suppressing EMT.

### Role of CTNNA1 in hepatic cancer

Targeted next-generation sequencing of 14 hepatocellular carcinomas (HCC) patients revealed molecular abnormalities that activate multiple pathways, including the Wnt pathway (CTNNA1 and CTNNB mutations) and the PI3K/AKT/mTOR pathway (mTOR, PIK3CA, and NF1 mutation) 78. It seems that abnormal CTNNA1 expression is associated with HCC. ATAD2 is highly expressed in HCC and is an independent prognostic factor that is positively correlated with HCC metastasis. MiR-372 is the upstream target of ATAD2 (an oncogene), and ATAD2 significantly upregulates APC and downregulates CTNNA1 by directly inhibiting its oncological effect 79. It is of interest to determine whether the miR-372-ATAD2-CTNNA1 axis plays a role in HCC. Many genes are upregulated in CD133^+^ cells of hepatitis C virus-associated HCC compared with normal controls, including CTNNA1, which may be responsible for the development and/or progression of the disease 80 (Fig. 3D).

### Role of CTNNA1 in other cancers

Downregulation or inhibition of CTNNA1 expression has been reported in osteosarcoma induced by the bone-seeking alpha emitter, 238Pu (which has been experimentally proven to induce the formation of bone tumors in animal osteosarcoma) 81, as well as in advanced thyroid cancer 82 and esophageal squamous cell carcinoma 83. The loss of both CDH1 and CTNNA1 is related mainly to follicular and anaplastic histology and lymph node metastases, with a biphasic pattern of α-catenin shown in thyroid cancer cases, correlating with the degree of differentiation of the examined malignancies (P = 0.01) 82. CTNNA1 interacts with Hras1-MAPK3 and Trp53 pathways in regulating the proliferation and apoptosis of skin cells using ultrasound-mediated rapid virus transfection in mouse embryos. Cutaneous cells with CTNNA1 deficiency are at a disadvantage in terms of cell proliferation. Excessive proliferation after CTNNA1 deletion is associated with downstream RAS-MAPK, and cell growth disadvantage after CTNNA1 deletion is associated with Trp53 activation (Fig. 3E) 84. The gene expression of high-risk HPV (human papillomavirus) infection-related esophageal cancer is different compared to that of low-risk HPV; for example, CTNNA1 expression decreases in esophageal cancer. Microarray data found that CTNNA1 expression is inhibited by HPV11 and HPV18 infection, and the inhibition is more obvious in HPV18 85. A single dose of pycnogenol induces significant apoptotic cell death in human fibrosarcoma cells *in vitro*. Microarray analysis showed that pycnogenol induced changes in the expression of various pathway genes in cancer cells, including the downregulation of CTNNA1, which is associated with increased survival of synovial sarcoma cells 86. Environmental differences also influence abnormal CTNNA1 expression. For instance, hypoxia induces downregulation of HIF expression and its regulatory proteins in lung cancer, including α-catenin 87. Immunohistochemical analysis of anaplastic lymphoma kinase (ALK) identified a fusion mutation of ALK-CTNNA1 in salivary gland secretory carcinomas 88. This fusion mutation is associated with the efficacy of specific cancer treatment (using tyrosine kinase inhibitor [TKI]), with an objective response rate of 85.7% with ALK-TKI in seven non-pulmonary solid tumors with CTNNA1-ALK or ITSN2-ALK fusion mutations for ALK (Table 2) 89.

## Discussion

Current reviews on the role of CTNNA1 in malignancies are limited mainly to HDGC 50, 53, 65, 90, 91; however, this review covers the pathogenesis of multisystem solid tumors and leukemia. In addition, there are well-established methods for CTNNA1 detection, such as qRT-PCR 73, immunohistochemistry 58, 73, exome sequencing 51, Sanger sequencing 64, and targeted next-generation sequencing 60. While new techniques, including intravenous injection of apatite carbonate nanoparticles carrying relevant proteins 29 or ultrasound-guided intrauterine fluorescence-traceable lentiviral techniques carrying RNAi or Cre recombinase 84, can help to track CTNNA1. Herein, we have fully summarized the current stage of CTNNA1 research methods, which can help to facilitate future studies in experimental design.

In many malignancies, CTNNA1 inhibits adhesion, invasion, and induces apoptosis of tumor cells by promoting or collaborating with CDH1 or inhibiting Wnt pathway. Current studies have identified reduced CTNNA1 expression in many types of tumors, including colorectal, breast and endometrial cancers. The reduced expression of CTRNNA1 is often accompanied by reduced expression of related proteins of cell adhesion complex E-cadherin-catenin, such as CDH1 and CTNNB1. For example, decreased expression CTNNA1 and CTNNB1 corresponds to the progression of *in situ* breast carcinoma to invasive carcinoma.

Mechanistically, CTNNA1 deletion has been shown to lead to an abnormal localization of E-cadherin and apoptosis resistance, resulting in cancer cells with invasive and anti-apoptotic properties. Knockdown of CDH1 leads to the reduced expression of CTNNA1 and CTNNB1 in endometrial epithelial cells, causing them to acquire invasive characteristics. CTNNA1 inhibited the transcription of genes related to tumor cell invasion by disrupting the binding of β-catenin-Tcf complex to DNA and suppressing the invasion of tumor cells. In collaboration with APC, CTNNA1 can promote the ubiquitination of β-catenin and inhibited the Wnt pathway. In addition, CTNNA1 can promote the metabolism of β-catenin by stabilizing APC, thereby inhibiting the Wnt pathway and suppressing cancer development. Future studies on CTNNA1 in malignancies should determine how CTNNA1 specifically affects the Wnt pathway and the E-cadherin-catenin complex, as well as its upstream targets, such as LSD1, PTEN and miRNAs.

CTNNA1 inhibits the metastasis of cancer by inhibiting EMT. Current findings have indicated that EMT contributes to tumor metastasis 92. CTNNA1 inhibits tumor metastasis and cell growth through inhibition of EMT in bladder cancer, and the reversal of the oncogenic effect of EMT by miR-429 and Setd2 in colorectal and pancreatic cancers is also associated with CTNNA1. Therefore, CTNNA1 inhibits tumor metastasis most likely, at least partly, through the inhibition of EMT. Furthermore, EMT is associated with metastasis in liver cancer 93, breast cancer 94, and endometrial cancer 95; however, no studies have demonstrated that EMT-related tumor metastasis in liver or breast cancer is associated with CTNNA1. The conclusion that CTNNA1 suppress cancer metastasis in bladder cancer via EMT provides novel insights into the role of CTNNA1 in many other carcinomas such as liver cancer, breast cancer, and endometrial cancer.

Hypermethylation of CTNNA1 is associated with shorter relapse-free survival in some malignancies. Current studies have observed that DNA methylation is widespread in aging cells and in various cancer 71. Many hematological malignancies showed histone hypermethylation (H3K27me3) at the promoter of CTNNA1. CTNNA1 hypermethylation is also present in endometrial and ovarian cancers, however the specific impact of CTNNA1 hypermethylation on progression and prognosis of these types of tumors remains unclear. The hypermethylation of CDH1 has been demonstrated in gastric cancer 71. As another member of E-cadherin-catenin complex, the methylation state of CTNNA1 in gastric cancer warrants further research.

At present, CTNNA1 mainly plays an essential role of inhibiting cell proliferation, promoting apoptosis, repressing invasion, and metastasis in malignancies. In some cancers, such as colorectal, bladder, breast cancers, the anti-cancer mechanism of CTNNA1 is well understood, which has been translated into clinical practice, such as screening CTNNA1 to guide the management of HDGC; however, in other cancers, such as endometrial, ovarian, and pancreatic cancers, the mechanism and related signaling pathway of CTNNA1 remain to be fully understood. Further studies will provide novel insights into the etiology and pathogenesis of more carcinomas, thereby improving treatment outcome.

## Figures and Tables

**Table 1:** Expression of CTNNA1 and its general effects in different malignancies

Cancer	Performance	Impact	References
Colorectal cancer	Downregulation	lymphatic metastasis; Differentiation; Prognosis; Invasion	9-11, 13
Breast cancer	Downregulation; Loss; Re-expression; Mutant	Metastasis	26, 27, 31
AML	Deletion; Hypermethylation	RFS	46
Therapy-related AML/MDS	Haploinsufficiency		39
Gastric cancer	Mutant	Comorbidity in FDR	55, 58, 61
Endometrial cancer	Downregulation	FIGO stage; Prognosis	67
Ovarian cancer	Hypermethylation	Progression-free survival	70
Bladder cancer	Downregulation	Pathological stage	72, 73
Pancreatic cancer	Upregulation	Metastasis	75, 77
Thyroid cancer	Downregulation	Differentiation; lymphatic metastasis	82
Esophageal cancer	Downregulation		83

**Table 2:** Impact of other factors on the expression of CTNNA1 in malignancies

	Function to CTNNA1	Cancer	References
238Pu	Downregulation	Osteosarcoma	81
HPV18	Downregulation	Esophageal cancer	85
Mir-200a	Downregulation	Esophageal cancer	83
Pycnogenol	Downregulation	Synovial sarcoma	86
Hypoxia	Downregulation	Lung cancer	87

## Abbreviations

CTNNA1: Catenin alpha 1
FAP: familial adenomatous polyposis
LOH: loss of heterozygosity
APC: adenomatous polyposis coli
CRC: colorectal cancer
EMT: epithelial-to-mesenchymal transition
CTNNAP1: pseudogene of CTNNA1
miR: microRNA
LCS: lobular carcinoma *in situ*
ILC: invasive lobular carcinoma
IDC: infiltrating ductal carcinoma
DCIS: ductal carcinoma *in situ*
RFS: recurrence-free survival
OS: overall survival
TCGA: The Cancer Genome Atlas
PARP: poly ADP ribose polymerase
MDS: myelodysplastic syndrome
AML: acute myeloid leukemia
CAN: copy number aberrations
TSG: tumor suppressor gene
MSP: Methylation-specific PCR
PTEN: Phosphatase and tensin homolog
C/EBP: CCAAT/enhancer binding protein
PRC2: polycomb complex 2
HDGC: hereditary diffuse gastric cancer
WHO: World Health Organization
NEEC: non-endometrioid endometrial carcinomas
HCC: hepatocellular carcinomas
HPV: human papillomavirus
ALK: anaplastic lymphoma kinase
TKI: tyrosine kinase inhibitor
